# Community-based nutrition-sensitive approach to address short-term hunger and undernutrition among primary school children in rural areas in a developing country setting: lessons from North and North-Eastern Uganda

**DOI:** 10.1186/s40795-020-00399-8

**Published:** 2020-12-14

**Authors:** Samuel Elolu, Duncan Ongeng

**Affiliations:** Department of Food Science and Postharvest Technology, Faculty of Agriculture and Environment, Gulu University, P.O Box 166, Gulu, Uganda

**Keywords:** Community, Nutrition innovation, School feeding, Hunger, Undernutrition

## Abstract

**Background:**

Undernutrition in childhood is an important factor that greatly impedes the achievement of full human potential at adulthood. Despite increased enrolment of pupils in primary schools in developing countries, short-term hunger and undernutrition continue to impact negatively on school attendance, retention and education outcomes in economically disadvantaged rural areas. This study examined the feasibility of a community-based participatory action research approach building capacity of rural women food vendors to use local food resources to produce nutritionally enhanced food products for primary school feeding in rural localities in a developing country setting.

**Methods:**

Mixed methods approach incorporating focus group discussions (FGDs) to evaluate parents’ and school administrators’ perceptions of the community-based approach, participatory experimental improvement of nutritional quality of an energy-based cassava product (gari) involving community women food vendors, and cross-sectional acceptability assessment of improved products among rural primary school children. Qualitative content analysis, one-way analysis of variance and correlation analysis was used to analyse FGD data, compare nutritional profile and consumer sensory profile of different products, and examine associations between sensory attributes and acceptability of the products, respectively.

**Results:**

The approach of using local food resources to produce nutritious products targeting school feeding was strongly recognised by parents, school administrators, teachers and small scale rural women food vendors as an adoptable nutrition-sensitive means of addressing short-term hunger among primary school children in rural settings. The action research resulted in a highly accepted nutritionally enhanced product (consisting of cassava, soy and silver fish) exhibiting superior nutritional properties (23.29% protein, 90.5 g/100 g calcium, 4.5 g/100 g zinc, 11.6 g/100 g iron, 40.40 g/100 g phosphorus, 61.57 μg/100 g vitamin A) compared to the original energy-dominated cassava product (2.18% Protein, 55.6 g/100 g calcium, 1.2 g/100 g zinc, 4.4 g/100 g iron, 6.6 g/100 g phosphorus, 11.23 μg/100 g vitamin A) (*p* < 0.05). Nutritional computation revealed that serving 120 g of the new product would suffice to meet 30% of the recommended dietary allowance for essential nutrients that children should receive from school meals.

**Conclusion:**

Community-level nutrition-sensitive innovation using local foods resources offers the opportunity for rural women food vendors to contribute to addressing short-term hunger and undernutrition challenges in primary schools in economically-disadvantaged localities in developing countries.

## Background

Inadequate nutrition in childhood has been recognized as one of the greatest impediments to fulfilment of full human potential. It affects over one billion people worldwide with the majority being in developing countries [1]. The central tenet of nutrition in human capacity development is based on the fact that deficiencies of essential nutrients at the childhood stage affect at an individual level, both the mental and physical status, resulting in poor health, poor educability, and poor work performance in adulthood [2]. The number of children enrolled in primary schools in developing countries increased tremendously over the past decades [3]. For instance, in Uganda, following the launch of the Universal Primary Education (UPE) program by the government in 1997, primary school enrolment increased from about three million children in 1996 to 8.3 million in 2009 and as of 2011 most primary-school-age children in the country attended school [4]. However, learner’s achievement levels, especially in resource-constrained rural areas such as north and north-eastern regions of the country have often been disappointing. Evidence on outcomes of national examinations over the last two decades indicates that pupils who studied and took examinations from schools in those regions have consistently had very poor performance [5–7]. Several reasons have been advanced to account for such a poor performance outcome. Among them are persistent short-term hunger and poor nutrition that collectively contributes to poor school attendance, inadequate cognitive development, and low educational attainment of the primary school learners.

A number of developing countries have initiated or put in place policies to support school feeding [8, 9]. Nonetheless, a critical look at existing policy frameworks from various countries in Africa reveals that most of them articulate the rationale and need to provide food to children while at school but are silent on the nutritional quality of the foods. In addition, although many school feeding policies mandate parents and or schools to ensure that children are fed at school, their implementation is not mandatory and the unintended implied consequence is that the better resourced and economically better-off localities easily implement while the under-resourced and economically disadvantaged localities are usually unable to do so. The use of locally available food resources to formulate nutritious food products is believed to be a potential strategy for addressing undernutrition challenges in resource-constrained localities in developing countries [4]. Indeed, substantial information on community-level research that used locally available food resources to develop elite nutritious composite products for complementary feeding of children in such localities in developing countries exists [10–12]. However, information is scarce on how a similar approach can be used to address short-term hunger and undernutrition experienced by primary school children in similar localities.

A review of literature on existing approaches to challenges of short-term hunger in primary schools in developing countries reveals that community input is largely not taken into account both in terms of development and use of results [13, 14]. This is notwithstanding the fact that literature within the realm of community development is awash with information to the effect that development outcomes of community-based initiatives are usually enhanced when the community is engaged in the process [15, 16]. From a nutritional point of view, it is apparent that most school diets in rural areas in developing countries if available are largely dominated by starchy staples (cereals and tubers) that mostly provide energy but are deficient in proteins and micronutrients. This situation calls for investment in easily accessible and adaptable low-cost nutritional innovations based on food resources available within the community to develop local capacity to respond to school feeding challenges in rural areas [4].

North and north-eastern Uganda are among the regions with the least developed rural areas, have the highest number of primary school drop-outs, and very low primary education performance in the country [7, 17]. These socio-economic characteristics make them a suitable geographical area for the development and testing of nutritional innovations to improve the nutritional welfare of primary school children in economically disadvantaged rural areas. Gari, a granulated cassava food product designed for making instant porridge has a high potential for application as cheap food for school feeding in economically disadvantaged rural areas such as those in the north and north-eastern Uganda. This is because its main raw material, cassava, is widely cultivated and consumed in those regions both as food security and a commercial crop. A major limitation associated with gari is that the product is largely energy-based but very low in protein (1.2%), deficient in essential amino acids and other micronutrients of public health importance such as calcium, iron, zinc, and phosphorous [18]. Therefore, without enrichment with other micronutrient and protein-rich sources, gari remains unsuitable for feeding school children as a base diet.

Therefore, using north and north-eastern Uganda as a case area, and gari as a base product for nutritional improvement, the objective of this study was to examine the feasibility of a community-based participatory action research approach, building the capacity of rural women food vendors to use local food resources to produce nutritionally enhanced products for application in primary school feeding in economically disadvantaged localities in a developing country setting. It is anticipated that local women food vendors would sell nutritious products in schools thus contributing to the nutrition well-being of primary school children as well as to the local economy of the area. In a much wider context, such an approach fits into the current agricultural development frontier which aims at making agribusiness nutrition-sensitive [19].

## Methods

### Focus group discussion

Eight Focus group discussions (FGDs) each were conducted among women food vendors, school administrators and teachers, and community members (parents) to: (i) assess the current status of school feeding in rural primary schools; (ii) determine perceptions of use of locally available food resources to produce food products tailored to nutrition needs of school-age children to be sold by local women food vendors in primary schools; and (iii) select food ingredients to be used for improving the nutritional quality of gari. Each focus group discussion had ten (10) members and for each, a set of discussion points covering the three general objectives identified above were interrogated. Discussions with parents and vendors were conducted using the local language while for school administrators and teachers the sessions were held in English.

### Development and production of nutritionally improved gari

**Fig. 1:**
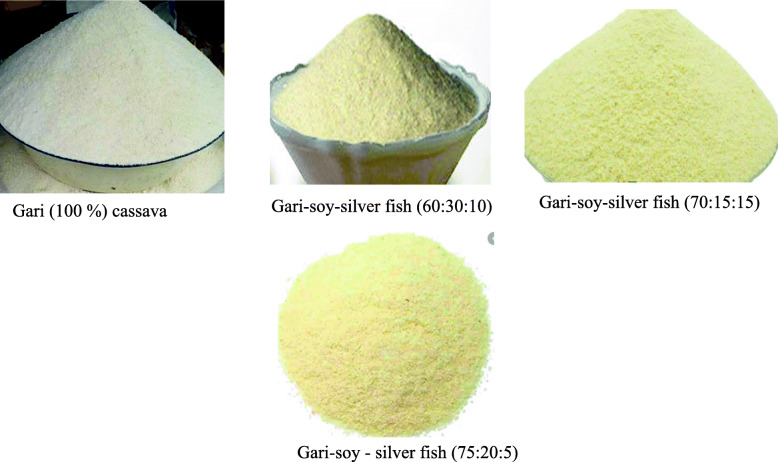
Pictures of gari formulated with various combinations of soy and silver fish (source: this study)

Using a participatory community engagement approach, local experience and expertise, and locally fabricated equipment, a method for producing nutritionally enriched *gari* at the community level was developed together with women food vendors at a farmer field school. The food vendors selected to participate were those who had at least 5 years of selling food in rural primary schools while those who were sick were excluded. On the basis of the FGD, soy and silver fish were selected as candidate resources for use in the nutritional improvement of gari. The selection of soy and silver fish was guided by the fact that they are locally available and are rich sources of protein and micronutrients. Soy is a complete protein source, rich in fat-soluble vitamins (A, D, E & K) and phosphorus (19), while silver fish is also a good source of proteins, minerals (e.g. calcium and phosphorus), and energy due to the high lipid content [20]. It has a balanced amino acid profile and is particularly rich in methionine and lysine that are deficient in soybean [21]. This implies that a combination of soybeans and silver fish, when used for gari fortification, allows for nutrient complementary benefits. Production of nutritionally improved gari followed three stages. First, raw soybeans and silver fish were separately roasted in a saucepan mounted on a charcoal stove consistent with the village practice in North and North-Eastern Uganda. Roasted materials were separately milled into a fine flour using a locally fabricated grain mill. In the second stage, fermented cassava cakes were produced following the method of Oluwamukomi [22], but with a modification to take care of local conditions. Briefly, fresh cassava tubers were peeled manually with a kitchen knife, washed, and grated using a locally fabricated mechanical grater. Grated cassava was packed in polythene bags and allowed to ferment for 24 h followed by pressing to dewater the mash. Wet cassava cakes were broken through a grater and pulverized. In the third stage, three gari composite formulae containing various levels of cassava, soybeans and silver fish (30% soy: 10% silver fish: 60% cassava; 15% soy: 15% silver fish: 70% cassava; and 20 soy: 5% silver fish: 75% cassava) were developed using Excel and Harvest Plus Food Composition Table [23]. For each composite formula, roasted soybean and silver fish flour were added at the determined ratios to the cassava mash and toasted to produce the nutritionally enhanced products. Figure 1 shows pictures of gari formulated with various combinations of soy and silver fish.

### Analysis of nutritional composition

Analyses were performed for moisture, dry matter, gross energy, total protein, ash, crude fibre, total carbohydrates, fats, phosphorus, calcium, iron, zinc, and vitamin A. Crude protein was determined using the Micro-Kjeldahl method as described by Magomya et al. [24]. Ash, gross energy, crude fat, moisture, dry matter, and total carbohydrates were determined according to the AOAC [25]. Vitamin A was quantified following the HPLC method previously described by Mieko et al. [26]. Phosphorus was determined following the vanado-molybdate colourimetry method while calcium, iron, and zinc were determined using the atomic absorption spectrophotometric method according to the AOAC [25].

### Sensory and acceptability evaluation among primary school children

Sensory evaluation was conducted to compare the level of consumer sensory preference and overall acceptability amongst the various gari products produced. The evaluation was conducted in 12 rural primary schools randomly selected by women food vendors and parents who participated in the FGDs. Consent was sought from parents of all pupils selected to participate in the study. Children with a history of an allergic reaction to soy (based on parent’s declaration) were excluded from the study. In addition, those who were sick were also excluded from the study. A total of 180 pupils in the upper primary (aged 10 to 14 years, corresponding with class level four to seven) were included in the evaluation. This is because pupils in this age category are expected to be capable of detecting differences compared to those in lower primary [27]. The samples were reconstituted into instant porridge with hot water according to Oluwamukomi [22]. Briefly, approximately 100 g of the test sample was added to 500 ml of hot water and stirred constantly to form a smooth thick paste. The samples were coded, randomized, and separately subjected to sensory evaluation by the selected primary school pupils. The pupils were provided with warm water for mouth rinsing before and after every sample tasting to eliminate carry-over effects [28]. The extent of liking of colour, texture, aroma, taste, and overall acceptability of the products was evaluated on a 5-point hedonic scale (ranging from 1 = poor to 5 = very good). This scale was purposely chosen for the study because it typically gives a higher response rate compared to other longer scales [29]. Besides, longer scales would be unsuitable for the primary school children who are non-experienced in sensory evaluation.

### Data analysis

Data from FGDs were analysed using mixed method content analysis [30]. This enabled the generation of a detailed description of qualitative information based on group consensus to understand the context of respondents’ perspectives towards school feeding. Essentially, the main themes emerging from different FDGs were identified and key findings summarised in a tabular form. In the case of nutritional improvement, One Way Analysis of Variance (ANOVA) was performed to compare the proximate composition, micronutrient contents, and scores for sensory preference and overall acceptability amongst various gari products. Correlation analysis was used to examine the association between sensory attributes (colour, aroma, texture, and taste) and overall consumer acceptability of the products. All statistical analyses were performed using SPSS version 2.0 while the level of statistical significance was fixed at 5%.

## Results

### Perceptions of women food vendors, school administrators, teachers, and parents

Results from the FGDs, summarised in (Table 1) indicate that parents, school administrators, and teachers strongly appreciated the need for school feeding. However, they also pointed out that such programs are largely lacking and in cases where they exist, it is not mandatory, thus the majority of pupils in the rural primary schools actually go through the school hours without any food. While the need to address the food needs of primary school children was dominantly pointed out, a specific understanding of nutrition with respect to limiting nutrients such as proteins and micronutrients did not emerge clearly from the parents as well as school administrators and teachers. In terms of bottlenecks, parents were largely constrained by the poor economic conditions in the area, lack of proper guidelines and laws to govern school feeding. Women food vendors were constrained by the lack of knowledge on food processing and nutrition needs of school children, lack of coordination with schools, and financial challenges. School administrators revealed that existing school feeding programs were initiatives of the schools, but their designs largely excluded parents’ participation. This notwithstanding, parents and women food vendors expressed a strong desire to cooperate with partners such as Universities and other institutions to design and test innovative approaches for school feeding. Specifically, parents viewed the proposed use of local food resources as a cheaper alternative to traditionally suggested school provisioning means that require parents to pay extra money for feeding. Generally, the idea of using cassava and other local food resources such as soy and silver fish as well as empowering local women food vendors as producers of nutritious foods for application in school feeding was well-received by all stakeholders that participated in the FGDs.

**Table 1:** Rural community perspectives on the status of school feeding

Themes of Discussion	Overall FGD findings
Overall status of school feeding	School-wide feeding programs are largely unavailable. Only parents who can afford to pack food or pay extra fees for feeding can have their children eat at school (these are very few). Previous school feeding interventions sponsored by Non-governmental organisations (NGOs) closed when projects ended. Nutritional quality of food is usually not taken into consideration in school feeding program designs thus meals are largely limited to corn-based porridge for breakfast.
The necessity of school feeding	There is consensus over the need to provide food to school-going children. It is strongly believed that providing meals will keep pupils at school and help them study better. The need for school food to meet the nutritional needs of children is well appreciated (considered important). However, nutritious food recipes tailored for application in school feeding in rural areas are largely unavailable.
Constraints to school feeding	Resources are inadequate (parents are unable to access adequate food at home as well as for school feeding) thus packed lunch is hard to afford. Foods previously provided and promoted by NGOs are expensive for parents while there are no clear guidelines and laws in place to enforce mandatory school feeding in rural schools.
Possible local community alternatives to school feeding	School gardening would be suitable since most schools in rural areas have farmland. Parents and school administration can also engage to produce food for school feeding. Local staple foods can be mobilised for school feeding from parents.
Knowledge of nutritional needs of school children and the improvement of local food resources	There is limited understanding of the nutritional quality of foods (current food provision focuses on supplying energy) by parents and women food vendors while teachers are aware of the nutrition needs of school children. No specific technologies are known or practised to improve the nutritional quality of local staple foods at community levels or in rural schools. Local community members lack knowledge on how to improve the nutritional quality of local foods.
Community-level partnerships for school feeding	Previously some NGOs such as World food program (WFP) and World Vision International have sponsored school feeding programs but with limited participation of the community. Thus NGO-led programs did not continue after NGO funding ceased. Existing school feeding initiatives are school-led, with limited involvement of parents and the general community in their design. The notion of active participation of the community in producing and processing food for school feeding programs is highly appreciated and is believed to ensure program ownership and sustainability.
Willingness to work with partners in the design and testing innovations on school feeding based on local food resources	Perceived as a good opportunity to gain knowledge and skills in nutrition interventions for local application. Use of local food resources would make school feeding more affordable for parents and rural schools. Building capacity in food processing would be a good business opportunity for the small scale women food processors and vendors to address the nutrition needs of school children.

Summary of information from eight focus group discussions (FGDs) with rural women food vendors, parents, and primary school administrators and teachers in north and north-eastern Uganda.

### Nutritional profile of the improved products

Proximate composition of the improved products in comparison with the original gari is presented in Table 2. Generally, the addition of soy and silver fish significantly improved the nutritional profile of gari. The formulations containing combinations of soy and silver fish had significantly higher levels of proteins, dietary fibre and fat (*p* < 0.05). Specifically, the protein content increased from 2.18% in pure gari to 23.29% in the gari-soy-silver fish composite while dietary fibre content increased from 5.63 to 10.53%. The fat content increased from 0.41 to 8.78% with increased soy and silver fish proportions leading to higher composite fat levels. Similarly, the addition of soy and silver fish significantly increased the ash content of gari (*p* < 0.05) from 1.41 to 4.22%. The rise in the level of ash increased with higher levels of soy and silver fish inclusion. On the other hand, the incorporation of soy and silver fish into gari significantly reduced the moisture (*p* < 0.05) and total carbohydrate content (*p* < 0.05) by about 41 and 42%, respectively. The reduction in carbohydrate content increased as the combined proportion of soy and silver fish in the formulation increased. Despite the reduction in carbohydrate content, gross energy increased by 22% per unit as a result of the addition of soy and silver fish.

**Table 2:** Proximate composition of gari formulated with various proportions of soy and silver fish

Composite Formulae	Crude Protein (%)	Total CHO (%)	Ash (%)	Dietary Fibre (%)	Moisture Content (%)	Fat content (%)	Gross energy (Kcal/g)
**Control (100:0)**							
	2.18 ± 0.00^**a**^	87.02 ± 1.86^**d**^	1.41 **±** 0.02^**a**^	5.63 **±** 0.17^**ab**^	8.66 **±** 0.13^**d**^	0.41 ± 0.56^a^	3.94 ± 0.03^**a**^
**Gari-Soy-silver fish**							
**60:30:10**	23.29 ± 0.42^**d**^	50.60 ± 1.25^**a**^	4.22 ± 0.08^**c**^	10.53 ± 0.62^**d**^	5.01 ± 0.07^**a**^	8.78 ± 0.16^c^	4.81 ± 0.01^**c**^
**70:15:15**	19.84 ± 0.39^**c**^	72.25 ± 1.84^**bc**^	3.91 ± 0.11^**c**^	6.88 ± 0.64^**bc**^	8.53 ± 0.12^**cd**^	5.64 ± 0.04^b^	4.34 ± 0.02^**b**^
**75:20:5**	15.10 ± 0.17^**b**^	72.39 ± 2.54^**bc**^	3.11 ± 0.00^**b**^	7.99 ± 0.30^**c**^	7.83 ± 0.11^**bc**^	5.69 ± 0.09^b^	4.38 ± 0.01^**b**^

Values are means ± SD of three independent determinations. Values with different superscripts within the same column are significantly different (*P* ≤ 0.05).

The contents of calcium, iron, zinc, phosphorus and vitamin A in the improved products in comparison to the original gari are presented in Table 3. In general, the inclusion of soy and silver fish significantly increased the mineral and vitamin A content of gari (*p* < 0.05). In all cases, the level of micronutrients in gari increased with increase in the proportion of soy and silver fish in the formulae. Specifically, the contents of calcium, iron, zinc, phosphorus, and vitamin A increased from 55.6 to 90.5, 4.4 to 11.6, 1.2 to 4.5, 6.6 to 40 g/100 g and 11.23 to 61.57 μg/100 g, respectively following the addition of soy and silver fish.

**Table 3:** Mineral content of gari formulated with different proportions of soy and mukene

Composite Formulae	Calcium (g/100 g)	Iron (g/100 g)	Phosphorus (g/100 g)	Zinc (g/100 g)	Vitamin A (μg/100 g)
**Control** (100:0)	55.6 ± 0.020^**a**^	4.40 ± 0.000^**a**^	6.60 ± 0.019^a^	1.20 ± 0.001^**a**^	11.23 ± 0.59^**a**^
**Gari-soy-silver fish**					
60:30:10	90.5 ± 0.020^c**d**^	11.60 ± 0.000^**c**^	40.40 ± 0.009^c^	4.50 ± 0.002^**d**^	61.57 ± 0.59^**d**^
70:15:15	86.7 ± 0.020^**c**^	10.40 ± 0.000^**b**^	39.50 ± 0.009^c^	3.90 ± 0.001^**c**^	54.67 ± 1.26^**c**^
75:20:5	83.3 ± 0.020^**b**^	9.80 ± 0.000^**b**^	22.70 ± 0.009^b^	2.30 ± 0.002^**b**^	41.79 ± 1.76^**b**^

Values are means + SD of three independent determinations. Values with different superscripts within the same column are significantly different (*P* ≤ 0.05).

### Consumer sensory preference and overall acceptability

Results of ratings of consumer sensory preference and overall acceptability of the improved products in comparison to the original gari are presented in Table 4. Generally, ratings for all the sensory parameters of formulations containing soy and silver fish were statistically similar to that of the original gari. Ratings for all the parameters were within acceptable limits with exception of the deviation indicating that pure gari (100% cassava) was best preferred in terms of colour. Thus, overall acceptability ratings were generally identical except for the formula containing 20% soy and 5% silver fish which was rated higher than the formulae containing other levels of soy and silver fish.

**Table 4:** Scores on sensory attributes and acceptability of gari by primary school pupils

Composite Formulae	Ratings for: Colour	Aroma	Texture	Taste	Overall acceptability
**Control (100:0)**	3.63 ± 1.37^**b**^	3.37 ± 1.27^**d**^	2.59 ± 3.09^**a**^	3.27 ± 3.09^**c**^	3.10 ± 1.30^**ab**^
**Gari-soy-silver fish**					
60:30:10	2.70 ± 1.44^**a**^	2.87 ± 1.38^**bc**^	3.02 ± 1.20^**b**^	2.93 ± 1.36^**abc**^	2.80 ± 1.30^**a**^
70:15:15	3.02 ± 1.37^**a**^	2.68 ± 1.25^**ab**^	3.33 ± 1.24^**b**^	2.85 ± 1.37^**ab**^	2.89 ± 1.26^**a**^
75:20:5	3.02 ± 1.34^**a**^	3.12 ± 1.27^**cd**^	3.20 ± 1.18^**b**^	3.13 ± 1.33^**abc**^	3.25 ± 1.30^**b**^

Values are means ± SD of scores by 180 pupils. Values with different superscripts within the same column are significantly different (*P* ≤ 0.05). Scores are based on a 5-point hedonic scale.

All the sensory properties had a strong positive correlation with overall acceptability irrespective of the formulation type (Table 5). In terms of magnitude, product taste had the strongest level of correlation with overall acceptability followed by aroma, texture and colour in decreasing order. However, despite the strong association with overall acceptability, none of the relationships was significant except in the case of original gari for which colour, texture and aroma significantly influenced overall acceptability. On the basis of the overall acceptability assessment, children chose the product with a combination of 75% cassava, 20% soy and 5% silver fish as the most preferred product and was consistent with the overall acceptability rating.

**Table 5:** Influence of sensory properties on overall acceptability of composites

Sensory Attribute	Gari (100:0)	Gari-soy-silver fish (60:30:10)	Gari-soy-silver fish (70:15:15)	Gari-soy-silver fish (75:20:5)
**Colour**	3.426	2.700	3.016	3.017
Acceptability	3.100	2.806	2.892	3.252
Correlation	0.301^a^	0.298^a^	0.190^a^	0.132
*P-value*	0.016	0.499	0.415	0.131
**Texture**	2.595	3.025	3.333	3.175
Acceptability	3.100	2.806	2.892	3.252
Correlation	0.330^a^	0.137	0.221^a^	0.398^a^
*P-value*	0.001	0.131	0.146	0.501
**Aroma**	3.369	2.867	2.680	3.118
Acceptability	3.100	2.806	2.892	3.252
Correlation	0.421^a^	0.220^a^	0.203^a^	0.299^a^
*P-value*	0.009	0.653	0.211	0.338
**Taste**	3.272	2.933	2.857	3.125
Acceptability	3.100	2.802	2.892	3.252
Correlation	0.300^a^	0.631^a^	0.628^a^	0.779^a^
*P-value*	0.592	0.204	0.685	0.145

Values with ^a^are statistically significant at 5% level of confidence.

## Discussion

The role of school feeding in tackling short-term hunger and undernutrition is critical in contributing to the positive outcome of education among children [31, 32]. Several approaches to school feeding exist [8, 33]. However, in the context of resource-constrained agrarian rural settings, developing linkages between school feeding and local agricultural development is generally viewed as a potential strategy for effecting school feeding programs. However, this strategy has not been well exploited in several developing countries [33, 34]. In this study, north and north-eastern Uganda was used as a microcosm for economically disadvantaged rural areas typical of a developing country setting. The fact that parents, rural women food vendors, school teachers and administrators had a strong positive perception towards commercially-oriented community-based school feeding approach suggests that the approach, whereas designed to address school feeding challenge, presents the opportunity to support the agricultural economy of the areas within the vicinity of the schools. One peculiar characteristic of this study was the participatory action research approach to the design, development and testing of the new nutritious products. The fact that stakeholders agreed and cooperated suggests that the approach offers a strong opportunity for local people to provide local solutions to school feeding challenge in economically disadvantaged rural areas. This is because the food resources used in the formulation of the nutritious product are locally available in the community.

Whereas rural women food vendors were enthusiastic about producing the developed nutritious products to support school feeding, it was apparent that they lacked technical knowledge and skills on how to formulate and produce nutritious products targeting nutrition needs of school children. This finding provides evidence to the effect that lack of sufficient knowledge on nutrition is an important factor that impedes judicial use of locally available food resources to address nutrition-related development challenges among economically disadvantaged agrarian communities in developing countries. By inference, therefore, it becomes apparent that building local capacity in nutrition-sensitive food processing linked to end-users of the products as illustrated in this study provides the opportunity for local investment in agri-food value addition to tackling local nutritional challenges.

Considering that cassava, the main raw material used in the production of the nutritious product is largely energy based [35], the introduction of soy and silver fish was meant to address the issue of deficiency of protein and micronutrients in cassava. It is important to appreciate that lack of inclusion of animal source foods in the diet is one of the factors that negatively affect the intake of bioavailable proteins and mineral micronutrients among economically disadvantaged communities in developing countries [36, 37]. This is because proteins and minerals from animal source foods are superior to those from plant sources because of low bio-availability from plant sources due to the presence of anti-nutritional factors [38, 39]. Therefore, it has been recommended that plant foods should be consumed together with animal foods to improve the bio-availability of proteins and minerals from the latter food group [38]. Additional advantages that formulations containing both soy and silver fish have are the amino acid complementarity benefit. For instance, soy protein is limited in sulphur amino acids while silver fish, an animal source protein is richer in amino acids methionine and lysine [21]. Additionally, soy contains significant amounts of bioactive compounds that are thought to offer protection against cardiovascular diseases and are believed to enhance brain and nerve functions [40]. This indicates the health benefits that gari containing soy would confer to school children. Whereas non-communicable diseases are more prevalent in ageing populations but less in children [41], it is probable that protection right from childhood age could reduce the chances of the problem occurring at old age. Thus, the use of soy and silver fish in the new cassava-based product does not only result in a nutritionally superior product but may also enhance the intake of bioavailable nutrients by school children as well as conferring other non-nutritional health benefits to them.

Appropriate dietary quality is an important factor that needs to be considered if school meals are to be in tandem with the recommended nutrient intake levels [9, 42]. A review of policy documents indicates peculiarity in nutritional standards for school meals among countries. For instance, many countries in South America have specifications for school meals set in the range of 20–30% of the daily nutrient requirements, while in Africa, specifications available for South Africa are set at 30% [9, 14]. However, information on the proportion of daily nutrient needs that school children should derive from school meals is largely lacking for the vast majority of developing countries including Uganda. Thus, basing on the South Africa specification, nutritional calculation reveals that serving 150 g of the new product would be sufficient to provide the total recommended daily protein requirement while 45 g would provide at least 30% of daily protein needs for children that school meals should provide. A similar calculation reveals that serving about 120 g of the enriched product would be sufficient for primary school children to meet 30% of the daily requirements for energy, as well as the recommended daily dietary allowance for calcium, iron, zinc and vitamin A. Nonetheless, a follow-up study focusing on the effect of the nutritious cassava product on nutrition outcomes among primary school children would provide more evidence on the potential of the product. Although the nutritional focus of product development reported in this study was on improving access to proteins and micronutrients of public health importance, it was also associated with changes in other important nutritional properties (such as carbohydrates, fat and energy content) of gari (Table 2). However, the most significant meriting attention was the increase in the fat content of gari. Considering the rich fatty acid profile of silver fish and soy, the increase in the fat content of the formulations would provide the opportunity for increased intake of essential fatty acids and omega-3 fatty acids in particular among school children. This is important considering that omega-3 fatty acids are important for cardiovascular health [43].

Consumer acceptability of food depends on a number on several factors including product sensory properties, familiarity, attractiveness and traditional values [44]. The fact that stakeholders (school administrators and teachers, parents and women food vendors) selected the food resources that constituted the new product suggests that issues of familiarity and tradition were favourable. Indeed, the high degree of enthusiasm with which women food vendors and primary school children participated in the study provides additional indications. As observed in previous studies [35, 45], in the current study, formulations containing higher proportions of soy had higher sensory ratings and were better preferred by the school children. This could be attributed to the pleasant aroma of roasted soy [35]. To the contrary, lower sensory scores were recorded for formulations with higher proportions of silver fish in terms of aroma and overall acceptability are likely due to the fishy odour from silver fish. This negative sensorial appeal is not peculiar to the product developed in this study. It was also encountered in fish fortified dairy products developed before [46]. Nonetheless, despite variations in the rating of sensory parameters among different formulations, the ratings for overall consumer acceptability were generally similar. This indicates that overall, improvement in the nutritional quality of gari through the incorporation of soy and silver fish at levels used in this study did not exert substantial negative sensorial deviations. Nonetheless, future studies should evaluate sensory stability of the nutritious products during storage to provide indications on the length of time they can remain acceptable.

The fact that there was a strong positive correlation between sensory properties of pure cassava-based gari and overall acceptance but not for other formulations containing soy and silver fish, suggests that overall acceptability of gari formulations containing soy and silver fish may be dependent on other non-sensory factors. This results also indicate that acceptability based on sensory ratings is not in tandem with the selection of better nutritious products. This observation further confirms the well-known fact that sensorial appeal overrides nutrition quality in determining food selection [47]. The nutrition-sensitive innovation presented in this paper successfully passed the development stage and is now ready for uptake by rural women food vendors and primary schools. However, due to the commercial-oriented nature of the product, its success in the school feeding market would largely depend on its affordability. A major limitation of this study however is that break-even analysis was not conducted and as such unit price and whether the product would be affordable is still unknown. Considering the significance of price in determining consumer willingness to pay for any commodity [48], future studies should therefore look at the economics of the nutritious cassava-based product in the market environment of rural primary schools. This analysis would be important because lack of resources was identified (during FGD) as one of the key impediments to parents’ support towards the school feeding programme.

## Conclusion

This study has demonstrated the potential of community-based action research in nutrition-sensitive value addition using local food resources to build local capacity to respond to short-term hunger and undernutrition among primary school children in economically disadvantaged rural areas in developing countries. Besides providing a local solution to the nutritional challenge faced by children in rural schools and offering an entrepreneurial opportunity to rural women food vendors, the study illustrates that community members are enthusiastic about participating in producing local research solutions to local challenges such as undernutrition and short-term hunger experienced by their children in rural primary schools. This approach offers an alternative means to the industrially fortified products that children in rural schools are usually unable to access.

## Data Availability

All relevant data from which conclusions of the manuscript have been drawn are presented in the paper.

## Abbreviations

FGD: Focus group discussion
UPE: Universal Primary Education
AOAC: Association of Official Analytical Chemists
HPLC: High Performance Liquid Chromatography
ANOVA: Analysis of Variance
SPSS: Statistical Package for the Social Sciences
NGO: Non-Governmental Organisation
WFP: World Food Program
GUREC: Gulu University Research Ethics Committee
RUFORUM: Regional Universities Forum For Capacity Building in Agriculture
